# Peripheral nerve‐blocks and associations with length of stay and readmissions in fast‐track total hip and knee arthroplasty

**DOI:** 10.1111/aas.14169

**Published:** 2022-11-28

**Authors:** Christoffer C. Jørgensen, Pelle B. Petersen, Louise O. Daugberg, Thomas Jakobsen, Kirill Gromov, Claus Varnum, Mikkel R. Andersen, Henrik Palm, Henrik Kehlet

**Affiliations:** ^1^ Section for Surgical Pathophysiology and the Center for Fast‐Track Hip and Knee Replacement Rigshospitalet Copenhagen University Copenhagen Denmark; ^2^ Department of Orthopedics Holstebro Hospital, Center for Fast‐Track Hip and Knee Replacement Copenhagen Denmark; ^3^ Department of Orthopedics, Interdisciplinary Orthopedics and the Center for Fast‐Track Hip and Knee Replacement Aalborg University Hospital Copenhagen Denmark; ^4^ Department of Orthopedics and the Center for Fast‐Track Hip and Knee Replacement Hvidovre University Hospital Copenhagen Denmark; ^5^ Department of Orthopedics and the Center for Fast‐Track Hip and Knee Replacement Lillebaelt Hospital – Vejle Copenhagen Denmark; ^6^ Department of Orthopedics and the Center for Fast‐Track Hip and Knee Replacement Gentofte Hospital Copenhagen Denmark; ^7^ Department of Orthopedics and the Center for Fast‐Track Hip and Knee Replacement Bispebjerg Hospital Copenhagen Denmark

**Keywords:** arthroplasty, enhanced recovery, ERAS, fast‐track surgery, hip, joint replacement, knee, nerve block, orthopedics, regional anaesthesia

## Abstract

**Background:**

Peripheral nerve blocks (PNB) have recently been recommended in total hip (THA) and knee (TKA) arthroplasty as they may reduce pain, morphine consumption, length of stay (LOS) and complications. However, whether PNBs are associated with early discharge within an enhanced recovery protocol including multimodal analgesia is uncertain.

**Methods:**

An observational multicenter study from January to August 2017 in six Danish Arthroplasty Centers with established fast‐track protocols. Prospective recording of preoperative characteristics and information on PNB, LOS and readmissions through the Danish National Patient Registry and medical records. Multiple logistic regression was used to investigate associations between PNB and a LOS >1 day, LOS >4 days, and 30‐days readmissions. We also reported on mobilization, pain, opioid and fall‐related complications leading to LOS >4 days or readmissions.

**Results:**

A total of 2027 (58.6%) THA and 1432 (41.4%) TKAs with a median LOS of 1 day (IQR 1–2) and 5.3% (CI:4.6–6.1) 30‐days readmission rate were identified. PNB was used in 40.7% (CI:38.2–43.3) of TKA and 2.7% (CI:2.0–3.5) of THA, but with considerable interdepartmental variation (0.0–89.0% for TKA). There was no association between PNB and LOS >1 day (OR:1.19 CI:0.82–1.72; *p* = .354), LOS >4 days (OR:1.4 CI:0.68–2.89; *p* = .359) or 30‐days readmissions (OR:1.02 CI:0.63–1.65; *p* = .935) in TKA. Logistic regression in THA was not possible due to limited use of PNB. In TKA there were 12 (2.1% CI:1.2–3.6) with and 1 (0.1% CI:0.02–0.7) without a PNB, who had mobilization, pain or opioid‐related complications, and 5 (0.9% CI:0.4–2.0) versus 4 (0.5% CI:0.2–1.2) who fell. Correspondingly, 2 (3.7% CI:1.0–12.6) and 11 (0.6% CI:0.3–1.0) of THA patients had these complications, while 0 (0.0% CI:0.0–6.6) and 17 (0.8% CI:0.5–1.3) fell.

**Conclusion:**

Routine use of peripheral nerve blocks was not associated with early discharge or 30‐days readmissions in fast‐track THA and TKA. Future studies should focus on benefits of PNB in high‐risk patients.

**Editorial Comment**

There is uncertainty whether peripheral nerve blocks reduce hospital length of stay (LOS) when included in fast‐track perioperative programs for knee or hip arthroplasty. This observational multicenter study involving 3459 patients tested for associations between peripheral nerve blocks and a hospital stay >1 day, >4 days, and 30‐days readmissions. Use of peripheral nerve blocks was not associated with longer postoperative hospital stay in this mixed cohort.

## 1 INTRODUCTION

Total hip and knee arthroplasty (THA/TKA) are increasingly common procedures and optimized perioperative care within an enhanced recovery program has led to major improvements regarding length of stay (LOS) and complications.^1^, ^2^ Despite this progress, major challenges still exist in order to improve pain‐management, patient satisfaction and functional outcomes.^3^, ^4^, ^5^ In this context, anesthetic and analgesic techniques may have an important role in reducing postoperative pain, and ultimately, improve recovery. The use of peripheral nerve blocks (PNB) has been much debated due to the analgesic effects resulting in reduced opioid consumption and improved patient satisfaction,^5^ but also because of potential reductions in LOS^6^, ^7^, ^8^ and various postoperative complications^5^ such as wound infections in TKA and pulmonary complications in THA.^6^ Subsequently, routine use of PNB has been recommended in several^9^, ^10^, ^11^, ^12^, ^13^ but not all recent guidelines.^14^

However, throughout the last years simple effective pre‐ and postoperative multimodal opioid‐sparing analgesia, including the additional analgesic effects of high‐dose preoperative steroids^15^ as well as high‐volume local anesthetic infiltration (LIA) in TKA,^16^ may have facilitated early recovery. Consequently, the need for routine use of PNBs to facilitate discharge to own home within the first postoperative days or reducing readmissions when applying an enhanced recovery or fast‐track protocol is uncertain. In fast‐track THA and TKA, continuous improvements achieving a median LOS of 1 day has been reported in a large detailed prospective multicenter consecutive cohort study of about 37,000 patients,^2^ but with limited knowledge on the use of PNBs. Thus, there is a lack of large‐scale investigations on the utilization of PNBs and potential benefits of standard use of PNBs within established fast‐track protocols. The present study aimed at investigating the use of PNBs and associations with LOS, readmissions and specific mobilization, pain and opioid‐related morbidity in six well‐established fast‐track THA and TKA centers with a median LOS of 1 day in 2017.^2^

## 2 METHODS

In Denmark observational studies are exempt from approval by the Regional Ethics committees, why no ethics approval was required. Permission to collect and store data was given by the Danish Data Protection Agency RH‐2007‐30‐0623 and permission to evaluate patient records without informed consent was given by the Danish Capital Region R‐20073405. The manuscript adheres to the STROBE‐guidelines and a study analysis plan was developed prior to data‐analysis.

We evaluated consecutively collected data from January to August 2017 in patients ≥18 years having primary elective THA/TKA in six dedicated fast‐track arthroplasty departments contributing to the Fast‐track Hip and Knee Replacement Center database (FCD). The FCD prospectively collects data on preoperative patient characteristics and hemoglobin in all patients scheduled for surgery at the respective departments.^2^ For the present study we excluded procedures due to fractures, cancer and severe congenital disorders. Patients with major lower extremity surgery within the previous 90 days were also excluded.

All departments have agreed to use similar fast‐track protocols including early mobilization, use of tranexamic acid, multimodal analgesia including pre‐incision high‐dose (125 mg) methylprednisolone and high‐volume local infiltration analgesia (LIA) in TKA, preferred spinal anesthesia and in‐hospital only thromboprophylaxis when LOS ≤5 days.^2^ Patients are discharged after achieving similar standardized functional discharge criteria with discharge to own home in >95% of cases.^2^, ^17^ However, the use of PNBs has not been standardized in the protocol but is utilized according to the preferences and expertise available at the individual hospitals.

In addition to the information from the FCD, all cases also had their medical records reviewed for information on use and type of PNB, type of anesthesia and use of LIA. Additional information on use of antihypertensives, antihyperglycaemics, anticoagulants, psychotropics and opioids within 3 months preoperatively was acquired from the Danish National Database on Reimbursed Prescriptions (DNDRP).^18^ Data was subsequently linked with the Danish National Patient Registry (DNPR) for data on LOS, readmissions and mortality. The DNPR registers >99% of all somatic admission in Denmark, regardless of location of surgery, ensuring complete 90‐days follow‐up.^19^ Any patient with a LOS >4 days and/or readmissions had their discharge summary reviewed with regards to reason for prolonged admission. In case of insufficient information in the discharge summary the complete medical record was reviewed. Any case of mortality had the entire medical record from both primary admission and/or readmissions reviewed to evaluate cause of death. Reviews were performed by PBP and complex cases were conferred with CCJ and HK until agreement was reached.

### 2.1 Statistical analysis

As there were no available data on potential effect size of PNB on LOS and readmissions in fast‐track THA and TKA, we were unable to conduct a pre‐study power analysis. Chi‐square and Fisher's exact test were used as appropriate with level of significance set at 0.05 and 95% confidence intervals for proportions calculated using http://vassarstats.net/. To investigate potential associations between use of PNB and LOS >1 day, LOS >4 days and 30 days readmissions we used a logistic regression model including all available preoperative characteristics, type of anesthesia and use of LIA as fixed effects and with department of surgery included as a random effect. Due to relatively few (<7%) cases with missing information on specific preoperative characteristics, anesthesia, use of LIA or PNB, we refrained from using multiple imputations and instead excluded cases with missing data from the logistic regression analysis. We had initially decided on analyzing both THA and TKA, but after evaluating data we decided to exclude THA from the logistic regression due to very few patients with PNB. Finally, we evaluated all complications resulting in LOS >4 days or 30‐days readmission due to pain, opioid‐side effects, lack of mobilization or falls in relation to use of PNB. Data was analyzed using SPSS v. 25. (IBM Armonk NY, USA).

## 3 RESULTS

**FIGURE 1:**
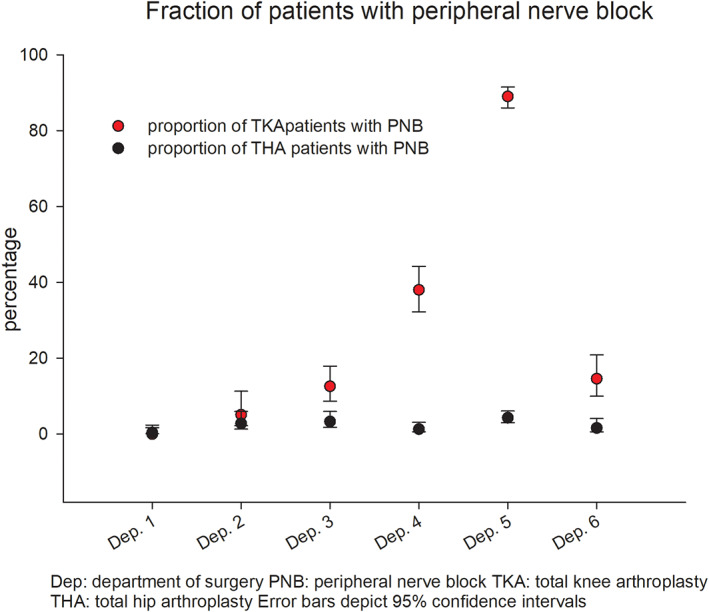
Proportion of patients with peripheral nerve blocks. Dep, department of surgery; PNB, peripheral nerve block; THA, total hip arthroplasty TKA, total knee arthroplasty. Error bars depict 95% confidence intervals.

We identified a total of 3471 procedures, but data on PNB was missing in 5 and 7 THAs. Thus, the final cohort consisted of 3459 procedures of which 2027 (58.6%) were THA and 1432 (41.4%) were TKA. Median LOS was 1 day (IQR 1–2) and with a 5.3% (CI:4.6–6.1) 30‐days readmission rate. Data on discharge destination was available in 2190 (63%) patients, of which 98.6% (CI:98.1–99.0) were discharged to own home. Spinal anesthesia was used in 78.8% (CI:77.4–80.1) of all procedures and LIA in 96.7% (CI:95.7–97.5) of TKA and 2.4% (CI:1.8–3.1) of THA. PNBs were used in 18.4% (CI:17.1–19.7) of all procedures, mainly after TKA (40.7% (CI:38.2–43.3)), but with considerable variation between departments (Figure 1).

### 3.1 TKA cohort

In TKA, the median LOS was 2 [IQR 1–2] and the fraction of patients with a LOS >1 day, LOS >4 days and 30‐days readmissions was 58% (CI:55–60), 5.6% (CI:4.5–6.9) and 5.0% (CI:4.0–6.3), respectively (Table 1). One department routinely used PNB (89.0% CI:86.0–91.5%) and consequently contributed with 74.4% of PNBs in TKA. In contrast, in the three departments using PNB second (38.0% CI:32.2–44.4) third (14.6% CI:10.0–20.9) and fourth (12.6% CI:8.7–17.9), PNBs were more common in patients having general anesthesia (70.0%, 35.0% and 77.3% of patients with general anesthesia, respectively). The remaining two departments rarely (5.1% CI:2.1–11.3) or never (0.0% CI:0.0–1.6) used PNBs. When adjusting for potential confounders and place of surgery, we found no association between PNB and LOS of >1 day (OR:1.19 CI:0.82–1.72; *p* = .354), LOS of >4 days (OR:1.40 CI:0.68–2.89; *p* = .359) or 30‐days readmissions (OR:1.02 CI:0.63–1.65; *p* = .935; Table 2).

**TABLE 1:** Characteristics TKA patients

n:1432 (41.4)	With PNB n: 583 (40.7)	Without PNB n: 849 (59.3)
Age mean (SD)	68.9 (9.7)	68.3 (9.7)
BMI mean (SD)	29.0 (5.3)	30.0 (5.4)
Missing	4 (0.7)	8 (0.9)
Gender		
Male	219 (37.6)	352 (41.5)
Female	364 (62.4)	497 (58.5)
Preoperative anaemia^a^	141 (24.2)	182 (21.4)
Missing	1 (0.2)	5 (0.6)
Use of walking aids	125 (21.4)	163 (19.2)
Missing	15 (2.6)	22 (2.6)
Social		
Living alone	213 (36.5)	291 (34.2)
With others	366 (62.8)	540 (63.6)
Institution	1 (0.2)	5 (0.6)
Missing	3 (0.5)	13 (1.5)
Smoking	66 (11.3)	101 (11.9)
Missing	1 (0.2)	12 (1.4)
Alcohol >24 g/day	55 (9.4)	65 (7.7)
Missing	1 (0.2)	13 (1.5)
Diabetes		
No diabetes	505 (86.6)	732 (86.2)
Diet treatment	6 (1.0)	15 (1.8)
Oral antidiabetics	62 (10.6)	76 (9.0)
Insulin dependent	8 (1.4)	15 (1.8)
Missing	2 (0.3)	13 (1.5)
Hypertension	336 (57.6)	511 (60.2)
Missing	0 (0.0)	0 (0.0)
Cardiac disease	79 (13.6)	122 (14.4)
Missing	4 (0.7)	16 (1.6)
Pulmonary disease	63 (10.8)	71 (8.4)
Missing	0 (0.0)	11 (1.3)
Psychiatric disorder	84 (14.4)	95 (11.2)
Missing	0 (0.0)	0 (0.0)
Anticoagulants	46 (7.9)	67 (7.9)
Missing	0 (0.0)	0 (0.0.)
Preoperative opioids	143 (24.5)	183 (21.6)
Missing	0 (0.0)	0 (0.0)
Anesthesia		
Spinal	381 (65.4)	731 (86.1)
General	189 (32.4)	106 (12.5)
General due to failed spinal	13 (2.2.)	12 (1.4)
Missing	0 (0.0)	0 (0.0)
Perioperative LIA	578 (99.1)	807 (95.1)
Missing	0 (0.0)	0 (0.0)
Median LOS (IQR)	2 (1–3)	2 (1–2)
30‐days readmissions	31 (5.3)	40 (4.7)
90‐days readmissions	51 (8.7)	55 (6.5)

^a^ Hemoglobin <12.9 g/dl.

Abbreviations: BMI, body mass index; IQR, interquartile range; LIA, local infiltration analgesic; PNB, peripheral nerve block; TKA, total knee arthroplasty.

**TABLE 2:** Results of multiple logistic regression analysis on association between peripheral nerve blockade and LOS >1, LOS >4, and 30‐days readmissions in TKA

Outcome	LOS >1 day		LOS >4 days		30‐days readmission	
	OR (95%CI)	*p* value	OR (95%CI)	*p* value	OR (95%CI)	*p* value
Peripheral nerve blockade	1.19 (0.82–1.72)	.354	1.40 (0.68–2.89)	.359	1.02 (0.63–1.65)	.935
Age	1.01 (0.99–1.02)	.472	1.07 (1.09–3.91)	.025	1.02 (0.99–1.05)	.198
BMI	1.01 (0.99–1.04)	.392	1.03 (0.98–1.08)	.254	1.03 (0.98–1.07)	.271
Male gender	0.51 (0.4–0.66)	<.001	0.67 (0.35–1.29)	.233	1.40 (0.83–2.34)	.208
Preoperative anemia	1.17 (0.869–1.57)	.304	1.42 (0.80–2.51)	.228	1.47 (0.87–2.48)	.148
Use of walking aid	1.73 (1.26–2.37)	.001	0.77 (0.41–1.45)	.420	1.60 (0.95–2.70)	.076
Living alone^a^	1.42 (1.10–1.84)	.008	1.31 (0.75–2.31)	.345	1.17 (0.71–1.93)	.534
Living in institution^a^	3.15 (0.32–30.7)	.322	4.24 (0.36–50.5)	.253	2.86 (0.29–27.82)	.365
Smoking	0.87 (0.6–1.26)	.466	1.79 (0.80–4.01)	.154	0.98 (0.47–2.07)	.096
Alcohol >2 units/day	1.32 (0.86–2.01)	.202	0,67 (0.19–2.35)	.527	0.94 (0.40–2.22)	.889
Diet treated diabetes	1.11 (0.43–2.87)	.829	3.55 (0.73–17.3)	.118	0.51 (0.04–6.82)	.611
Oral antidiabetics	1.07 (0.71–1.60)	.760	1.09 (0.48–2.44)	.839	1.01 (0.48–2.12)	.978
Insulin dependent	3.73 (1.16–11.9)	.027	4.95 (1.29–18.98)	.020	7.45 (2.64–20.98)	<.001
Hypertension	0.87(0.69–1.16)	.402	1.71 (0.90–3.25)	.103	0.86 (0.52–1.44)	.575
Cardiac disease	1.03 (0.67–1.55)	.884	1.22 (0.57–2.62)	.606	0.89 (0.41–1.93)	.760
Pulmonary disease	1.02 (0.69–1.52)	.935	1.49 (0.69–3.22)	.316	1.89 (1.01–3.53)	.045
Psychiatric disorder	1.00 (0.69–1.43)	.979	2.07 (1.09–3.91)	.025	1.44 (0.79–2.63)	.235
Anticoagulants	1.08 (0.64–1.81)	.786	1.26(0.49–3.27)	.632	0.95 (0.36–2.55)	.924
Preoperative opioids	1.02 (0.77–1.36)	.879	1.32 (0.75–2.35)	.338	1.42 (0.86–2.34)	.171
Spinal anesthesia	0.71 (0.52–0.98)	.035	0.59 (0.32–1.08)	.084	0.83 (0.48–1.45)	.509
Local infiltration analgesia	1.15 (0.60–2.21)	.665	2.81 (0.36–22.18)	.328	1.04 (0.29–3.74)	.951

*Note*: A total of 87 patients were excluded to missing data on specific variables, thus the final logistic regression models include 1345 patients with department included as a random effect.

Abbreviations: BMI, body mass index; CI, confidence interval; LOS, length of hospital stay; TKA, total knee arthroplasty.

^a^ Compared with living with others.

The majority of PNBs in TKA were adductor canal (ACB) or saphenous nerve blocks (94.2% CI:92.0–95.8), while the use of catheters with continuous infusions was extremely limited (1.4% CI:0.6–2.6; Table 3). In 34 patients (6.8% of patients with PNB) a subsequent PNB was provided, either the same day or the day after. There was a total of 13 (0.9% CI:0.5–1.6) TKA patients with a LOS >4 days or 30‐days readmission due to either pain, opioid side‐effects, or lack of mobilization. Of these 12 (2.1% CI:1.2–3.6) had a PNB and 1 (0.1% CI:0.02–0.7) did not. Finally, there were 9 (0.6% CI:0.3–1.2) falls, of which 5 (0.9% CI:0.4–2.0) were in TKA patients with and 4 (0.5% CI:0.2–1.2) in TKA patients without a PNB.

**TABLE 3:** Types of peripheral nerve blocks

Procedure	THA 52 (2.6)	TKA 583 (40.8)
Type of block		
Saphenous nerve/adductor canal adductor canal catheter	10 (18.6) 0 (0.0)	541 (92.8) 8 (1.4)
Popliteal sciatic nerve	0 (0.0)	2 (0.4)
Cutaneous lateral femoral nerve	24 (44.4)	0 (0.0)
Femoral nerve	15^a^ (31.5)	11 (1.9)
Details not available	3 (5.5)	20 (3.5)

*Note*: *n* (%).

Abbreviations: THA, total hip arthroplasty; TKA, total knee arthroplasty.

^a^ A further two cases of PNB were applied due to periprosthetic fractures either during or in the day after the primary procedure.

### 3.2 THA cohort

In THA, the median LOS was 1 [IQR 1–2] day, while 43% (CI:41–45), 4.7% (CI:3.9–5.7) 5.5% (CI:4.6–6.5) had a LOS of >1, LOS of >4 days or 30‐days readmission, respectively (Table 4). The number of PNBs was limited to 52 cases (2.6% CI:2.0–3.5). The department with the most PNB after THA had used these in 28 (4.3% CI:3.0–6.1) of cases and was also the one using standard PNB after TKA. The remaining departments used PNBs in 0.4–3.3% of THA. The main types of PNBs were femoral nerve and lateral cutaneous femoral nerve (29.6% and 44.4%, respectively) (Table 3), and three patients (5.6% CI:1.9–15.1) had a subsequent PNB. Due to the limited number of THA patients with a PNB we refrained from adjusted analysis, but univariate analysis found PNB to be associated with LOS >1 day (OR:1.5 [CI:1.2–1.9] *p* < .001) and LOS >4 days (OR:2.3 [CI: 1.4–3.6] *p* < .001), but not with 30‐day readmissions (OR:1.1 [CI:0.7–1.8] *p* = .604).

**TABLE 4:** Characteristics THA patients

n: 2027 (58.6)	With PNB n: 52 (2.6)	Without PNB n: 1975 (97.4)
Age mean (SD)	63.3 (15.3)	69.1 (10.9)
BMI mean (SD)	27.5 (4.9)	27.1 (4.7)
Missing	1 (1.9)	14 (0.7)
Gender		
Male	23 (44.2)	786 (39.8)
Female	29 (55.8)	1189 (60.2)
Preoperative anaemia^a^	18 (34.6)	478 (24.2)
Missing	0 (0.0)	9 (0.5)
Use of walking aids	10 (19.2)	491 (24.9)
Missing	3 (5.8)	34 (1.7)
Social		
Living alone	25 (48.1)	1217 (61.6)
With others	25 (48.1)	727 (36.8)
Institution	2 (3.8)	11 (0.6)
Missing	0 (0.0)	20 (1.0)
Smoking	12 (23.1)	258 (13.1)
Missing	0 (0.0)	19 (1.0)
Alcohol >24 g/day	2 (3.8)	144 (7.3)
Missing	0 (0.0)	17 (0.9)
Diabetes		
No diabetes	47 (90.4)	1779 (90.4)
Diet treatment	2 (3.8)	27 (1.4)
Oral antidiabetics	2 (3.8)	119 (6.0)
Insulin dependent	1 (1.9)	34 (1.7)
Missing	0 (0.0)	16 (0.8)
Hypertension	27 (51.9)	1033 (52.3)
Missing	0 (0.0)	0 (0.0)
Cardiac disease	6 (11.5)	263 (13.3)
Missing	1 (1.9)	23 (1.2)
Pulmonary disease	10 (19.2)	179 (9.1)
Missing	0 (0.0)	14 (0.7)
Psychiatric disorder	15 (28.8)	259 (13.1)
Missing	0 (0.0)	0 (0.0)
Anticoagulants	3 (5.8)	162 (8.2)
Missing	0 (0.0.)	0 (0.0)
Preoperative opioids	21 (40.4)	524 (26.5)
Missing	0 (0.0)	0 (0.0)
Anesthesia		
Spinal	14 (26.9)	1540 (78.0)
General	36 (69.2)	401 (20.3)
General due to failed spinal	2 (3.8)	31 (1.6)
Missing	0 (0.0)	3 (0.2)
LIA	11 (21.2)	37 (1.9)
Missing	0 (0.0)	1 (0.1)
Median LOS (IQR)	2 (1–3)	1 (1–2)
30‐days readmissions	3 (5.8)	107 (5.4)
90‐days readmissions	4 (7.7)	151 (7.6)

Abbreviations: BMI, body mass index; IQR, interquartile range; LIA, local infiltration analgesic; THA, total hip arthroplasty; PNB, peripheral nerve block.

^a^ Hemoglobin < 12.9 g/dl.

A total of 13 (0.6% CI:0.04–1.1) THA patients had a LOS >4 days or 30‐days readmission due to either pain, opioid side‐effects, or lack of mobilization. Of these 2 (3.7% CI:1.0–12.6) had a PNB and 11 (0.6% CI:0.3–1.0) did not. There was a total of 17 (0.8% 0.5–1.3) falls, of which none were in patients with PNB (0.0% CI:0.0–6.6).

## 4 DISCUSSION

In this descriptive multicenter study on use of PNBs in elective fast‐track THA and TKA, we found that about 18% of patients received an intra‐ or postoperative PNB. The use of PNBs was highly dependent on place of surgery and having TKA, but not on preoperative patient characteristics. Finally, in TKA patients we found no association between PNB and LOS or readmissions when adjusting for preoperative patient characteristics and choice of anesthesia, nor with specific pain or fall‐related complications. As this was a multicenter study in unselected patients and covering most of the Danish health‐care regions, we believe that our results are broadly applicable and relevant for everyday practice in most centers with established enhanced recovery/fast‐track protocols.

PNBs have previously been suggested to improve postoperative outcomes after THA and TKA, including reduction in pain, opioid consumption, LOS, emergency department visits, readmissions and hospital costs.^6^, ^7^, ^9^, ^10^ Thus, a Canadian propensity‐matched population based study in TKA found that while nerve catheters with continuous infusions increased LOS, single shot PNBs reduced LOS by 0.1 day and with reduced risk of readmissions.^7^ However, the study relied purely on diagnostic codes and administrative claims data between 2002 and 2014, and reported a mean LOS of 4.8 days with no considerations on LIA, type of multimodal analgesia or whether readmissions where related to the surgical procedure. Finally, the used PNBs were different from those in our study (mainly femoral nerve and fascia iliac blocks^7^ versus ACB and saphenous nerve blocks in the present study). This may merely reflect a change in anesthetic practice but could also suggest less focus on early mobilization as femoral and fascia iliac nerve blocks may have an undesirable effect on motor function. Interestingly, the authors found that the association with reduced LOS was reversed when focusing only on continuous PNBs and after 2008, where PNBs were associated with increased LOS. This supports our findings of no association between PNB and short LOS in modern fast‐track practice.

Another, retrospective population‐based study in >1,000,000 THA and TKAs between 2006 and 2013 found that several postoperative complications, especially wound complications in THA and pulmonary complications in TKA, were reduced when using PNBs. Furthermore, they found a reduction in median LOS from 4 to 3 days with a PNB.^6^ However, this study was also based on administrative claims data and with no data on use of LIA, type of PNB, use of an enhanced recovery protocol, or specific readmission data.^6^

A recent consensus statement from the International Consensus on Anesthesia Related Outcomes after Surgery group (ICAROS) based on a thorough systematic review and meta‐analysis found that most postoperative complications, especially cardiac, respiratory, and renal complications were reduced in both THA and TKA patients receiving PNBs. As there were no apparent increase in any complications, they recommend use of PNBs unless contraindications are present, or expertise is unavailable.^12^ However, although planned, a sensitivity analysis investigating the potential mitigating effects of an implemented enhanced recovery program was not possible. Neither was investigation of the effect of individual types of PNB or LIA, possibly due to lack of sufficient data. Interestingly, the work of the ICAROS group suggested that the effect of PNB was consistently stronger in groups having general anesthesia^12^ which was only used in about 20% of our patients. Consequently, the recommendation of routine use of PNB may not be applicable in a fast‐track setup with preferred spinal anesthesia, LIA in TKA and a multi‐modal analgesic regimen including high‐dose glucocorticoid, as in our cohort.

The two recent updates of the procedure specific postoperative pain management group (PROSPECT) guidelines, which also focus on use of multimodal analgesia and enhanced recovery, now recommend routine LIA combined with ACB after TKA and fascia iliac blockade after THA. In TKA this recommendation is based on an equal or potentially greater analgesic effects, particularly on dynamic pain, compared with LIA alone.^13^ However, the combination of ACB and LIA was only found to provide superior pain control during the initial 24 h and with no opioid sparing effect compared with by itself LIA.^13^

While superior pain control the first 24 h may facilitate early discharge, we found no association with use of PNB and discharge on postoperative day 1 in the TKA cohort. Furthermore, our data did not support increased readmission rates in patients without a PNB due to pain or lack mobilization within the initial 30 days after surgery. In THA,^11^ the PROSPECT group recommends LIA and a fascia iliac blockade in all patients, but especially in patients with expected high postoperative pain or in case of contraindications to basic analgesics. This recommendation is partly based on three different meta‐analyses published in 2019 finding reduced pain and morphine consumption, no increase in falls and potentially reduced LOS with use of fascia iliac blockade.^11^ However, yet another meta‐analysis published in 2021, did not find that a fascia iliac block had any effect on those parameters.^20^ In our study, the THA population had a median LOS of 1 day despite no fascia iliac blocks and only a few received LIA, reflecting current clinical practice. Also, use of a PNB actually increased the risk of LOS >1 day, although this must be interpreted with caution due to few PNB's prohibiting meaningful adjusted analysis. Nevertheless, our data do not support that routine use of LIA or PNB are required in fast‐track THA.

The strengths of our study include the use of a recent consecutive unselected cohort of patients with a well‐defined fast‐track protocol including multimodal analgesia and complete follow‐up, and with the only major difference being the utilization of PNB.^2^ Also, the combination of patient‐reported preoperative characteristics with national registry data on opioid use, and specific information on LIA and type of PNB, provides information mostly unaccounted for in previous large database studies.^6^, ^7^ Furthermore, we used discharge summaries and health records reasons investigating reasons for LOS >4 days and readmissions, thus enabling specific analysis on morbidity potentially related to the use of PNBs.

Limitations of our study include potential confounding by indication and unmeasured confounding. Thus, although our results do not suggest routine use of PNBs is needed to achieve a short LOS or reduce readmissions, it is not possible to draw any conclusion with regards to the use of PNBs in patients having general anesthesia, as rescue analgesic techniques or in “high‐pain” responders or patients with chronic pain.^21^, ^22^ In this context, a randomized study‐design would be more suitable for evaluating the benefits of PNB in such patients, whereas the large‐scale observational design in our study may be more appropriate for evaluating general effects in a real‐life clinical setting.

Also, as with any chart review, our data on reasons for LOS >4 days or readmissions are susceptible to inaccuracies in registration and subjectivity regarding interpretation. Especially the lack of information on the level of regional anesthetic expertise of the anaesthesiologist who performed the blockade or whether all relevant nerves, for example, the nerve to Vastus Medialis in case of ACB after TKA, were targeted may be problematic as it would influence block success rates. However, few database studies include such data^6^, ^7^ and we believe that the use of information from the electronic health records is preferable compared with singular use of claims‐data which are unintended or suboptimal for scientific research. Finally, we focused mainly on LOS and readmissions which, although being common outcomes in several studies on use of PNB^6^, ^7^, ^8^ may not only depend on use of a PNB. Thus, we did not have sufficient data to explore other highly relevant outcomes of regional anesthetic success, such as pain intensity, opioid consumption, or patient satisfaction.^4^, ^5^

In conclusion, our study did not find routine use of peripheral nerve blocks to be associated with early discharge or fewer 30‐days readmissions after fast‐track THA and TKA. Future studies should focus on PNB in certain high‐risk patients including those with expected high levels of postoperative pain.

## FUNDING INFORMATION

The study was supported by a grant from Candys Foundation. The Candys Foundation had no influence on development of the study analysis plan or any aspect of data‐collection, data analysis or interpretation of the results.

## CONFLICT OF INTEREST

Prof. Kehlet and Dr. Gromov are advisory‐board members of “Rapid Recovery” by Zimmer Biomet. Dr. Varnum has received travel reimbursement from Stryker. The remaining authors have no potential conflicts of interest.

## ETHICS STATEMENT

In Denmark observational studies are exempt from approval by the Regional Ethics committees, why no ethics approval was required. Permission to collect and store data was given by the Danish Data Protection Agency RH‐2007‐30‐0623 and permission to evaluate patient records without informed consent was given by the Danish Capital Region R‐20073405.

## Data Availability

Anonymized data is available from the authors upon request.
